# Ipsilateral inhibitory rTMS restores interhemispheric motor cortex balance and reduces phantom limb pain: an exploratory longitudinal nTMS mapping study

**DOI:** 10.1186/s12984-026-02120-5

**Published:** 2026-08-13

**Authors:** Ghaith Altawalbeh, Eugen Ursu, Filippo Emanuele Colella, Maria Goldberg, Raimunde Liang, Axel Schröder, Sebastian Ille, Sandro M. Krieg, Melissa Zavaglia, Amartya Ganguly, Chiara Negwer, Bernhard Meyer, Arthur Wagner

**Affiliations:** 1 Department of Neurosurgery, Klinikum rechts der Isar, School of Medicine, Technical University of Munich (TUM), Ismaninger Str. 22, 81675 Munich, Germany; 2 Department of Biomedical Sciences, Humanitas University, Milan, Italy; 3 Department of Neurosurgery, Heidelberg University Hospital, Heidelberg, Germany; 4 Munich Institute of Robotics and Machine Intelligence (MIRMI), Technical University of Munich (TUM), Munich, Germany

**Keywords:** Phantom limb pain, Repetitive transcranial magnetic stimulation, Interhemispheric imbalance, Cortical reorganization, Navigated TMS, Motor mapping, Amputation, Callosal disinhibition

## Abstract

**Background:**

Phantom limb pain (PLP) is a debilitating condition associated with maladaptive neuroplastic changes following limb amputation. The prevailing neuromodulatory approach targets the deafferented contralateral motor cortex, yet interhemispheric imbalance is increasingly recognized as a central pathophysiological feature of PLP. We investigated whether inhibitory repetitive transcranial magnetic stimulation (rTMS) applied to the ipsilateral (unaffected) hemisphere could reduce PLP and induce measurable normalization of interhemispheric cortical asymmetry.

**Methods:**

Two individuals with traumatic right upper-limb amputation and severe PLP underwent a longitudinal study comprising a four-week baseline phase, an inhibitory 1 Hz rTMS intervention targeting the ipsilateral motor cortex, and a five-week follow-up phase. Serial navigated transcranial magnetic stimulation (nTMS) motor mapping of both hemispheres was performed before and after the intervention. A principal component analysis (PCA)-based spatial projection and Gaussian kernel regression pipeline was developed to quantify motor map area, center of gravity (CoG), mean motor evoked potential (MEP) amplitude, and signal amount bilaterally.

**Results:**

Both participants demonstrated clinically meaningful PLP reductions (66% and 33% relative decreases on the Visual Analog Scale [VAS]), sustained across the five-week follow-up. Serial nTMS mapping revealed enlarged residual limb motor representations in the affected hemisphere at baseline. Following rTMS, cortical area of the triceps representation in the affected hemisphere decreased by 67% and 61% in the two participants respectively, with concurrent normalization of interhemispheric differences in both representation area and signal amount. CoG shifts were modest (mean 3.28 mm), indicating focal map consolidation rather than wholesale topographic reorganization.

**Conclusions:**

These findings offer novel neurophysiological evidence suggesting that inhibitory rTMS targeting the ipsilateral motor cortex reduces PLP while concurrently normalizing interhemispheric motor map asymmetry. The results are consistent with a callosal disinhibition mechanism, whereby ipsilateral inhibition may facilitate adaptive reorganization of the deafferented hemisphere. The novel PCA-based bilateral mapping pipeline introduced here provides a quantitative framework for future controlled trials.

## Background

Phantom limb pain (PLP) affects between 30% and 85% of persons with amputation, depending on amputation level, etiology, and assessment methodology [1–3]. In persons with traumatic upper-limb amputation — a population that tends to be younger and otherwise healthy — prevalence estimates typically range from 30 to 60% [4, 5]. PLP is characterized by the perception of pain in a limb that no longer exists, frequently described as burning, cramping, or shooting in quality. Despite considerable advances in understanding its pathophysiology, PLP remains refractory to many conventional treatments, and chronic PLP substantially impairs quality of life [6].

The primary sensorimotor cortex undergoes substantial reorganization following limb amputation [7, 8]. Classical maladaptive plasticity accounts link PLP to invasion of the deafferented hand area by neighboring body-part representations and to medial shifts of motor and somatosensory maps that correlate positively with pain intensity [9–11]. More recent persistent representation models emphasize preserved structure and function of the former hand cortex alongside reduced inter-regional connectivity, suggesting that maintained phantom representations and altered network dynamics may equally underlie chronic PLP [12]. Critically, both frameworks converge on interhemispheric imbalance and disrupted sensorimotor network dynamics as key, but incompletely understood components of PLP pathophysiology.

Under physiological conditions, the primary motor cortices (M1) of each hemisphere maintain dynamic equilibrium via transcallosal inhibitory circuits. Unilateral limb loss disrupts this balance through permanent deafferentation and asymmetric cortical reorganization, resulting in altered interhemispheric inhibition (IHI) and excitability [13–15]. Expanding cortical representations of residual limb muscles in the affected hemisphere and concurrent changes in the unaffected hemisphere have been documented [16], underscoring that post-amputation plasticity is a bilateral phenomenon.

Transcranial magnetic stimulation (TMS) is a non-invasive technique for probing and modulating cortical excitability. When applied repetitively (rTMS), it can shift excitability in a frequency-dependent manner: low-frequency stimulation (≤ 1 Hz) generally reduces excitability at the stimulated site [17], while simultaneously modulating the contralateral hemisphere via transcallosal pathways. This callosal disinhibition principle — inhibiting the unaffected hemisphere to disinhibit the affected one — has been exploited therapeutically in stroke and neurosurgical postoperative rehabilitation [18–20]. Navigated TMS (nTMS) additionally enables precise, MRI-guided coil positioning and serial motor mapping, allowing quantification of cortical reorganization across time. Two recent systematic reviews have synthesized evidence on rTMS for PLP [21, 22], and with the sole exception of a single case report [23], all included studies applied stimulation exclusively to the contralateral (deafferented) hemisphere. Di Rollo and Pallanti (2011) first described inhibitory 1 Hz rTMS targeting the ipsilateral hemisphere and reported pain reduction in one participant; however, that study did not assess cortical plasticity changes, leaving the underlying neurophysiological mechanism entirely uninvestigated.

The present study addresses this gap directly. We introduce a novel quantitative framework combining inhibitory ipsilateral rTMS with serial bilateral nTMS motor mapping, PCA-based spatial analysis, and Gaussian kernel regression heatmaps to characterize longitudinal cortical reorganization in two participants with traumatic upper-limb amputation and severe PLP. Our central hypothesis is that inhibitory rTMS applied to the ipsilateral M1 reduces IHI directed toward the deafferented hemisphere — a callosal disinhibition mechanism — thereby facilitating adaptive reorganization and reducing PLP. Beyond clinical outcomes, this study introduces a replicable neurophysiological mapping pipeline that provides a quantitative foundation for future sham-controlled trials of ipsilateral rTMS in PLP.

## Methods

### Ethics

The local ethics board reviewed and approved the study protocol (Ethical Committee Reference Number: 2023-591-S-KH). All procedures were conducted in accordance with the Declaration of Helsinki. Written informed consent was obtained from each participant prior to study enrollment.

### Participants

Two male participants (Participant 1 [P1] and Participant 2 [P2]) who sustained traumatic right upper-limb amputation were enrolled. Both sustained amputation at the mid-humerus level: P1 with a residual stump of approximately 15 cm (6 months post-amputation at study inclusion) and P2 with a residual stump of approximately 10 cm (18 months post-amputation). Neither participant used a prosthesis. Both were receiving pregabalin at the time of enrollment and reported persistent severe PLP primarily localized to the missing hand region, described as burning and cramping in quality. Baseline PLP intensity averaged 7.96/10 for P1 and 7.00/10 for P2 on the Visual Analog Scale (VAS) during the four-week baseline phase. Prior to the baseline phase, P1 discontinued pregabalin entirely, while P2 reduced his dosage from 100 mg ([1, 2, 2]) to 0-0-2 (Table 1). Comprehensive neurological examination confirmed preserved proximal motor function, including shoulder anteversion, retroversion, and abduction. Magnetic resonance imaging (MRI) of the brachial plexus and residual limb was performed to exclude neuroma formation, infection, or structural plexus lesions.

**Table 1:** Baseline demographic and clinical characteristics

participant	Age (years)	Months since amputation	Indication	Amputation site	Stump length	Pregabalin at baseline	Mean PLP VAS at baseline (0–10)
1	36	6	Traumatic	Right upper arm	15 cm	No	7.96
2	23	18	Traumatic	Right upper arm	10 cm	Yes, 100 mg 0-0-2	7.00

*PLP: phantom limb pain; VAS: Visual Analog Scale*

### Study design

**Fig. 1:**
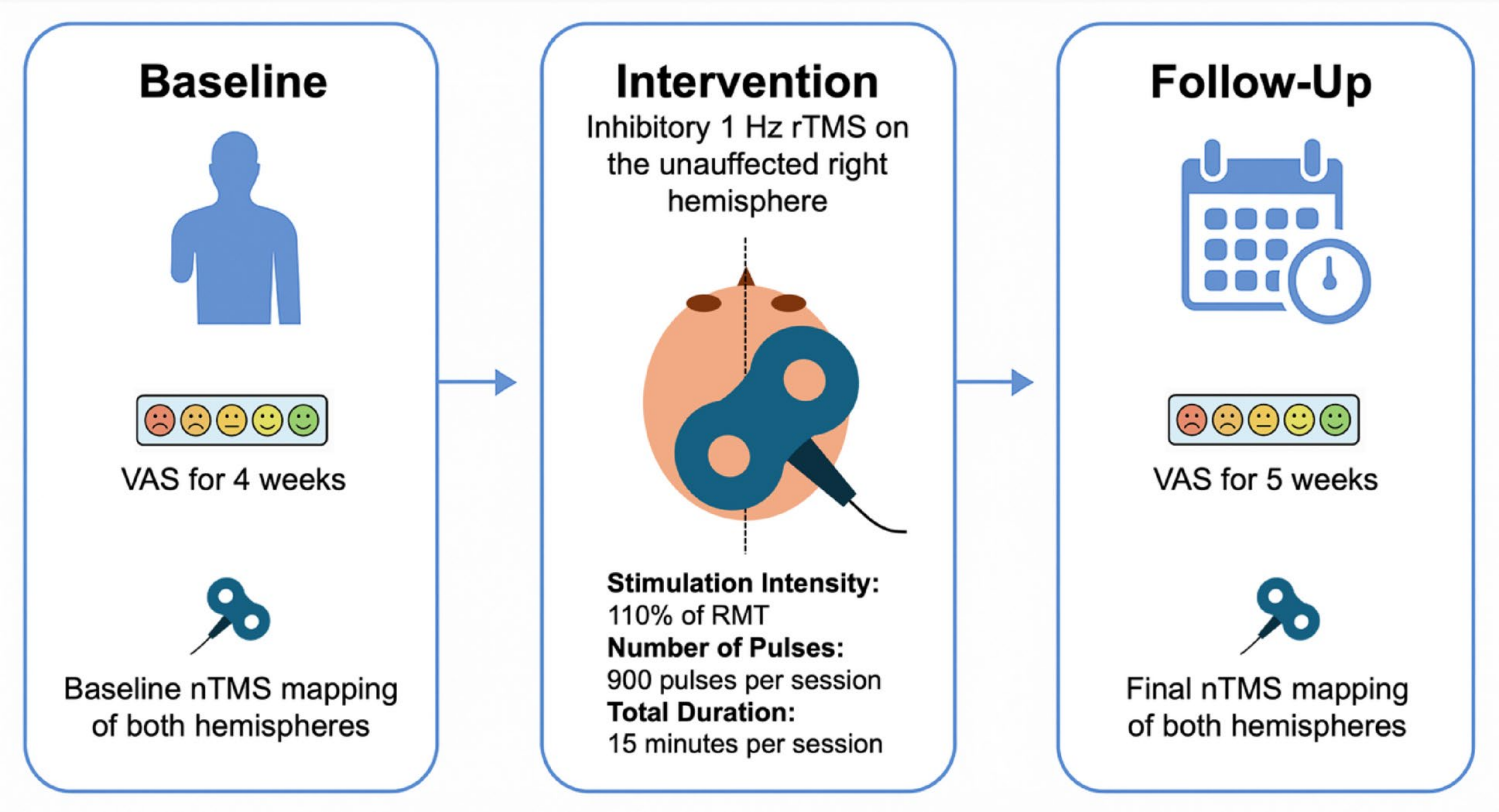
Study design. Three sequential phases: (1) four-week baseline with bilateral nTMS mapping and weekly VAS assessment; (2) rTMS intervention applying inhibitory 1 Hz stimulation (900 pulses per session) to the ipsilateral (unaffected) M1; (3) five-week follow-up with daily VAS and post-intervention bilateral nTMS mapping. *nTMS*: Navigated transcranial magnetic stimulation; *VAS*: Visual Analog Scale; *M1*: Primary motor cortex; *rTMS*: Repetitive transcranial magnetic stimulation

**Fig. 2:**
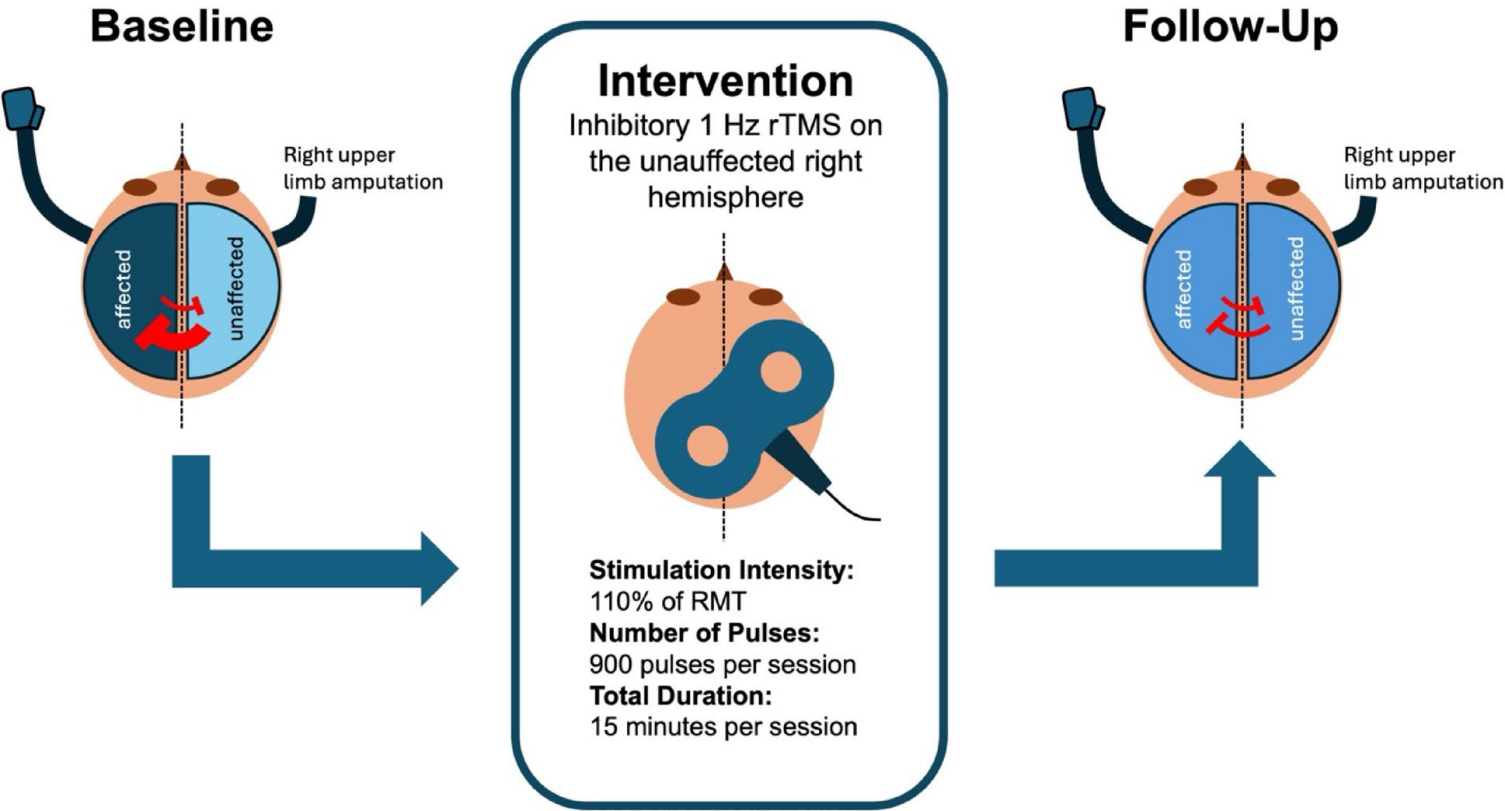
Hypothesized callosal disinhibition mechanism. Schematic illustrating baseline interhemispheric imbalance, with excessive inhibitory drive directed from the unaffected toward the affected hemisphere, and the proposed effect of ipsilateral inhibitory rTMS in reducing this inhibitory drive and permitting consolidation of the affected hemisphere’s motor representation. *M1*: Primary motor cortex; *rTMS*: Repetitive transcranial magnetic stimulation

The study comprised three sequential phases (Fig. 1): (1) a four-week baseline phase with bilateral nTMS motor mapping and weekly VAS pain assessment; (2) an intervention phase consisting of inhibitory 1 Hz rTMS targeting the ipsilateral (unaffected, right) motor cortex; and (3) a five-week follow-up phase with daily VAS assessment and a post-intervention nTMS mapping session. The hypothesized callosal disinhibition mechanism motivating this design is illustrated in Fig. 2.

All mapping sessions were conducted using a neuronavigation system (Nexstim Navigated Brain Stimulation System 5 [NBS System 5]; Nexstim Plc, Helsinki, Finland) guided by a high-resolution 1 mm-slice anatomical MRI enabling three-dimensional brain reconstruction and precise coil positioning. Surface electromyographic (EMG) recordings were obtained bilaterally from three innervated residual limb and intact contralateral limb muscles: biceps brachii (BIC), triceps brachii (TRI), and deltoid (DEL). Bipolar electrode configurations were used following standardized procedures for reliable motor evoked potential (MEP) detection [24].

A motor hotspot and resting motor threshold (RMT) were determined separately for each of the three recorded muscles (BIC, TRI, DEL) in each hemisphere at each session, using the 5/10 method: the minimum stimulator intensity at which ≥ 5 of 10 consecutive stimuli evoked MEPs ≥ 50 µV peak-to-peak [24]. The 5/10 method was chosen over the system’s automated adaptive threshold-estimation algorithm because it allowed the experimenter, specifically trained in this criterion, to visually inspect each trace in real time and reject artifactual or volitionally contaminated responses, which a fully automated algorithm does not always permit; MEPs were accepted only after visual confirmation of a quiescent pre-stimulus EMG baseline, with no pre-innervation activity or tonic background contraction. Single-pulse TMS mapping of each muscle was conducted at 110% of that muscle’s own RMT, using the neuronavigation system’s integrated stimulation grid (inter-target spacing approximately 5 mm, three suprathreshold pulses per grid point), proceeding systematically medially and laterally along the central sulcus until MEP responses were absent, with coil orientation maintained perpendicular to the sulcus throughout. Both hemispheres were mapped following this identical protocol, so that bilateral comparisons were not confounded by procedural differences between hemispheres. Directional labels used elsewhere in this manuscript (e.g., antero-superior) refer to the native anatomical coordinate system of each participant’s MRI reconstruction within the neuronavigation software, with the superior axis oriented toward the superior sagittal sinus and the anterior axis toward the frontal pole.

Inhibitory rTMS was delivered at the triceps hotspot of the ipsilateral (unaffected) hemisphere at 1 Hz, 110% of the triceps RMT, with 900 pulses per session (15 min). Sessions were scheduled on consecutive weekdays (Monday–Friday), one session per day, for up to five planned sessions per participant, informed by comparable rTMS designs in the PLP and chronic pain neuromodulation literature [21, 22]. Given enhanced cortical plasticity expected during the earlier post-amputation period, P2 (18 months post-amputation) completed all five planned sessions, whereas P1’s (6 months post-amputation) sessions were adaptively stopped after the second session once a clinically meaningful reduction in PLP was observed, since pain relief was the primary clinical endpoint.

### TMS data analysis and visualization

TMS data were exported from Nexstim sessions as session-specific coordinate tables for each arm. Stimulation coordinates were recorded in native MRI space (mm). Each stimulation point was linked to EMG responses from the corresponding muscles and quantified by the peak-to-peak amplitude of the MEP (µV).

To enable consistent spatial comparison across sessions, hemispheres, and participants, a principal component analysis (PCA)-based dimensionality reduction was applied. For each participant and each arm, stimulation points were pooled across all sessions and projected onto a dedicated 2D PCA space. Coordinates from individual sessions were centered and projected into this space without scaling, preserving metric units in mm. Where necessary, custom rigid transformations (rotation and reflection matrices) were applied to align maps across hemispheres and individuals.

Motor activation maps were generated using kernel regression with a 2D isotropic Gaussian kernel (bandwidth h = 1 mm). Values outside the convex hull of stimulation points were set to zero. Iso-contours at 50 µV were overlaid on all activation maps.

$$\:f\left(x\right)=\frac{{\sum\:}_{i=1}^{N}{K}_{h}(x-{x}_{i}){z}_{i}}{{\sum\:}_{i=1}^{N}{K}_{h}(x-{x}_{i})}$$

$$\:{K}_{h}(x-{x}_{i})=\frac{1}{2\pi\:{h}^{2}}exp(-\frac{\parallel\:x-{x}_{i}{\parallel\:}^{2}}{2{h}^{2}})$$

$$\:Z=ME{P}_{amplitude}$$

The center of gravity (CoG) was computed as the amplitude-weighted centroid of all suprathreshold (> 50 µV) grid points following smoothing.

$$\:{x}_{com}=\:\frac{{\varSigma\:}_{Z>50\:uV}x\cdot\:z}{{\varSigma\:}_{Z>50\:uV}z},{\:y}_{com}=\:\frac{{\varSigma\:}_{Z>50\:uV}y\cdot\:z}{{\varSigma\:}_{Z>50\:uV}z}$$

Cortical representation area was defined as the total area (mm²) of the region exceeding 50 µV. Signal amount — an integrated measure of cortical output — was computed as the product of mean MEP amplitude and area for suprathreshold points, equivalent by the intermediate value theorem to the integral of smoothed activity above threshold.

## Results

### Clinical outcome: phantom limb pain

During the four-week baseline phase, mean VAS scores were 7.96/10 (P1) and 7.00/10 (P2), quantified through weekly averages of daily assessments. Following rTMS, both participants demonstrated clinically meaningful PLP reductions sustained across the entire five-week follow-up, operationalized per the Initiative on Methods, Measurement, and Pain Assessment in Clinical Trials (IMMPACT) consensus criteria as a decrease of ≥ 2 points or ≥ 30% on the 0–10 VAS scale [25].

**Fig. 3:**
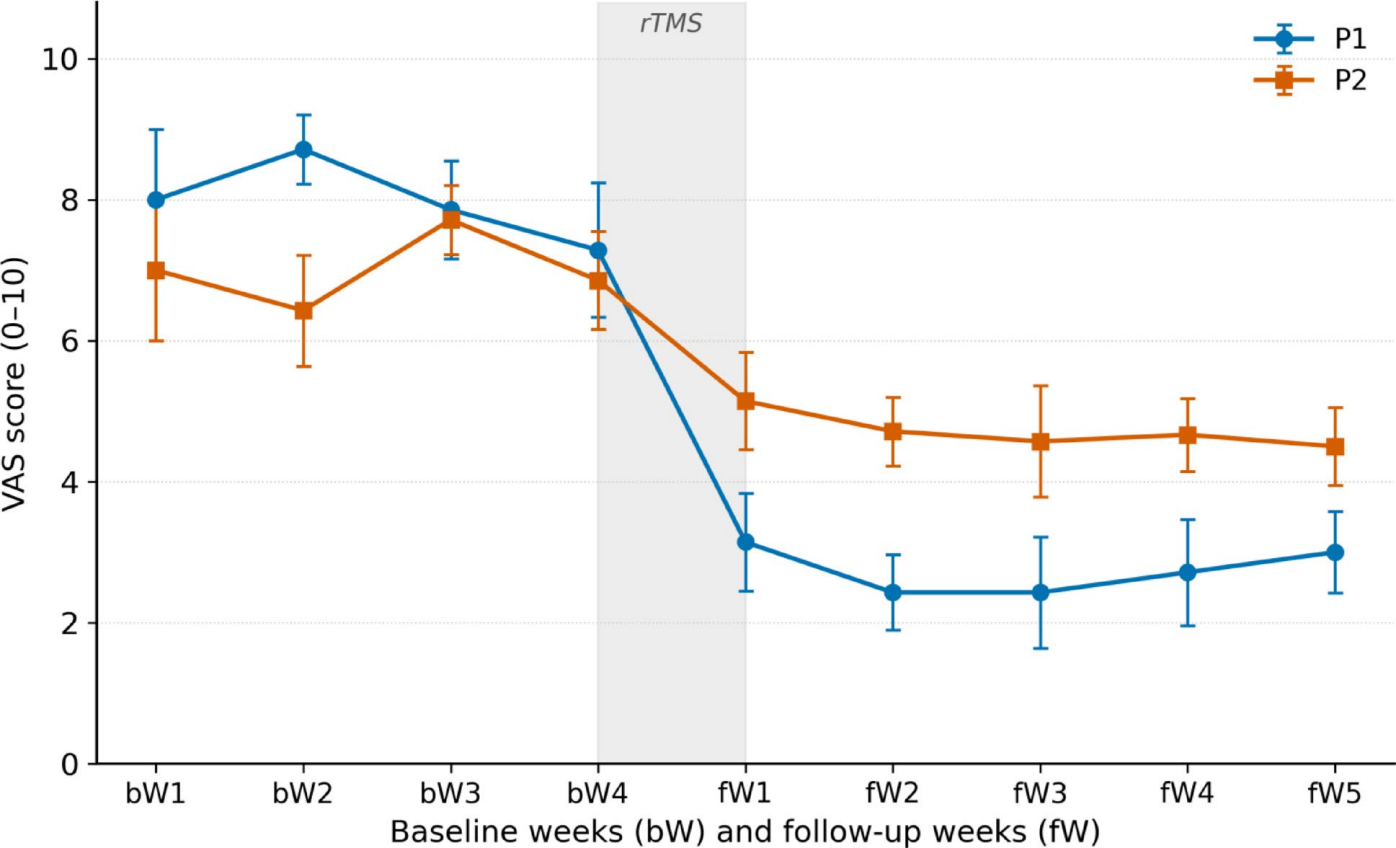
Averaged weekly VAS scores during baseline and follow-up. Weekly averages of daily self-reported VAS scores for P1 and P2 during baseline weeks (bW1–bW4) and follow-up weeks (fW1–fW5).* VAS: *Visual Analog Scale;* P1: *Participant 1;* P2: *Participant 2;* rTMS: *Repetitive transcranial magnetic stimulation

Post-intervention mean VAS scores were 2.74 for P1 and 4.72 for P2, representing absolute reductions of 5.22 points (66% relative reduction) and 2.28 points (33% relative reduction), respectively. P1’s improvement exceeded the IMMPACT threshold for substantial clinically important change (≥ 50% reduction or ≥ 4 points), while P2’s reduction met the criterion for moderately important change (≥ 30% or ≥ 2 points). Pain reduction was temporally consistent throughout the follow-up interval, indicating sustained rather than transient analgesic efficacy (Fig. 3).

### Cortical reorganization: quantitative nTMS analysis

Bilateral nTMS mapping was performed before and after the rTMS intervention, with EMG recordings from BIC, TRI, and DEL. TRI was selected as the primary outcome muscle on the basis of consistent, robust MEPs across all sessions in both participants, whereas BIC and DEL recordings were frequently confounded by pre-activation and tonic background EMG activity from postural stabilization of the residual limb, increasing trial-to-trial variability and complicating reliable threshold and amplitude estimation for these muscles; focusing the primary quantitative analysis on TRI also improves the clarity and interpretability of the reported results given the constraints of an *N* = 2 case series. Data for BIC and DEL are presented for descriptive completeness. Quantitative assessments encompassed CoG location and shift magnitude, cortical representation area (> 50 µV), mean MEP amplitude, and signal amount.

### Affected (left) hemisphere: baseline to follow-up

**Fig. 4:**
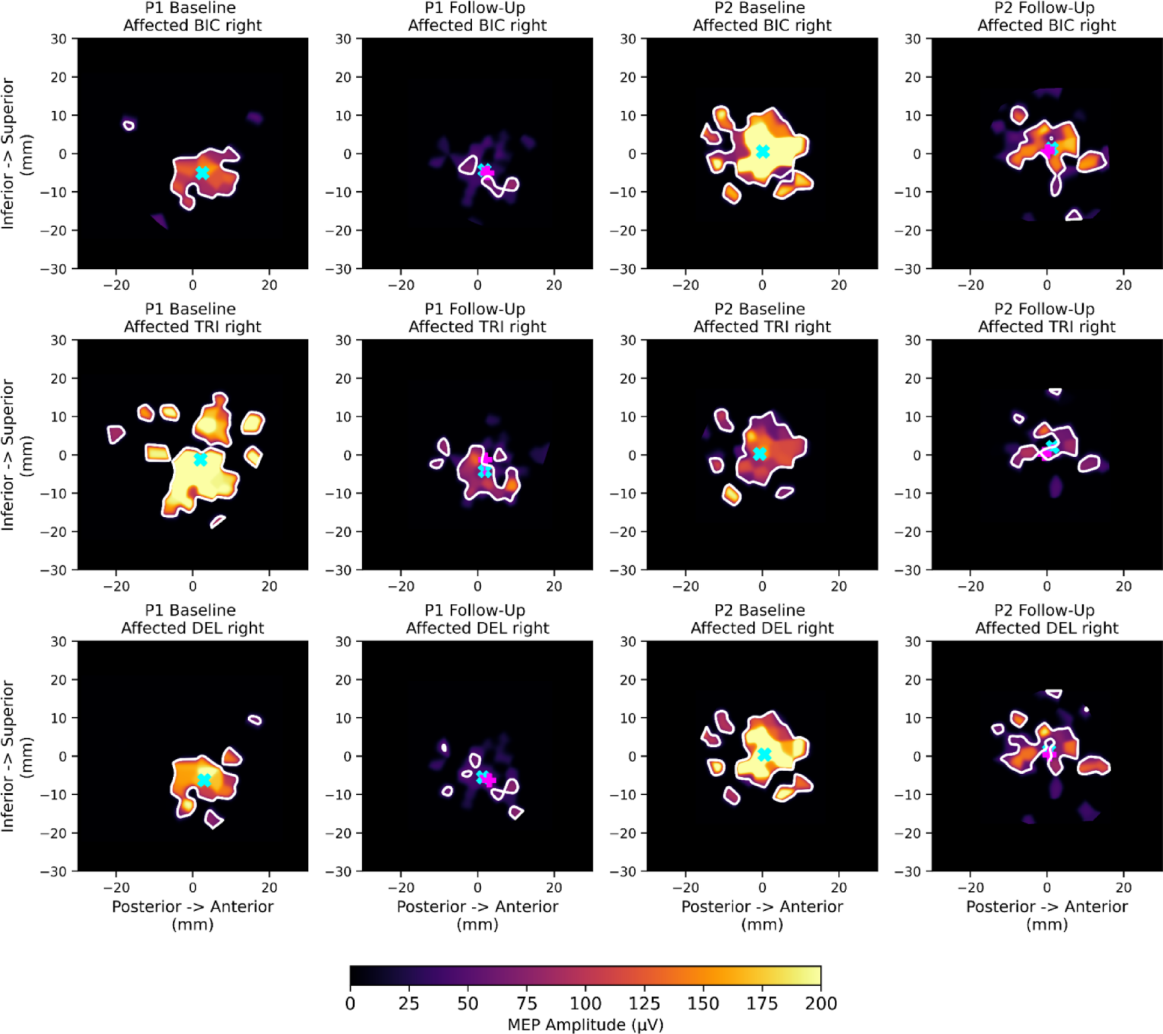
Cortical excitability maps in the affected (left) hemisphere. Gaussian kernel-smoothed MEP activation maps for BIC, TRI, and DEL at baseline and post-rTMS follow-up for P1 and P2. The x- and y-axes correspond to PCA-derived physical dimensions in millimeters (range −30 to 30 mm), maintaining a consistent spatial frame across participants and sessions. The 50 µV iso-contour (white line) demarcates the representation boundary. Each map shows the smoothed MEP amplitude distribution using a perceptually uniform colormap ranging from 0 µV (black) to 200 µV (white). Current session* CoG: *Turquoise X*; Baseline CoG in follow-up maps: *Purple X*. MEP: *Motor evoked potential;* BIC: *Biceps brachii;* TRI: *Triceps brachii*; DEL: *Deltoid; *CoG: *Center of gravity;* PCA: *Principal component analysis;* mm: *Millimeters

At baseline, both participants exhibited markedly enlarged triceps motor representations in the affected hemisphere relative to the unaffected side (Fig. 6; see Interhemispheric Comparison section below). Following rTMS, compression of the cortical representation area was observed across all three muscles (BIC, TRI, DEL) in both participants (Fig. 4). CoG shifts were modest for all muscles, indicating focal consolidation rather than wholesale spatial reorganization.

Quantitatively, P1 demonstrated a 67% reduction in triceps area (from 416.48 to 137.59 mm²) with a concurrent 53% reduction in mean MEP amplitude, and a CoG shift of 3.75 mm in an antero-inferior direction. P2 showed a 61% area reduction (from 254 to 98.26 mm²), a 59% reduction in mean MEP amplitude, and a CoG shift of 2.81 mm postero-superiorly (Table 2).

**Table 2:** Triceps representation in the affected (left) hemisphere

Parameter	P1 (6 months post-amputation)	P2 (18 months post-amputation)	Mean
Baseline Area (mm²)	416.48	254.00	335
Follow-Up Area (mm²)	137.59	98.26	118
Area Reduction (mm²)	278.89	155.74	217
Area Reduction (%)	66.96	61.31	64
CoG Shift Magnitude (mm)	3.75	2.81	3.28

*CoG: center of gravity; mm²: square millimeters*

#### Unaffected (right) hemisphere: baseline to follow-up

**Fig. 5:**
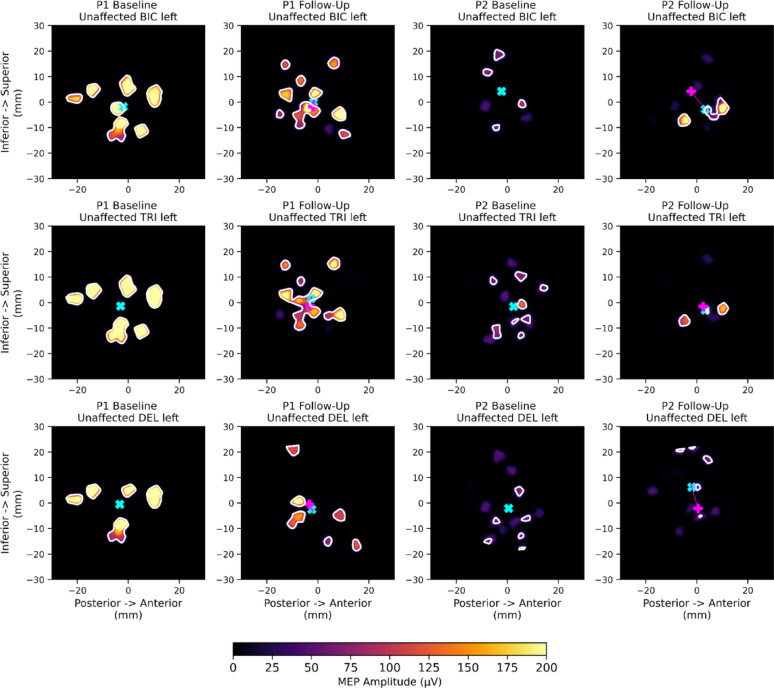
Cortical excitability maps in the unaffected (right) hemisphere. Format identical to Fig. 4. Reductions in triceps representation area at the rTMS stimulation site are visible in both participants

As the direct target of inhibitory rTMS, the unaffected right hemisphere showed measurable reductions in triceps cortical excitability in both participants (Fig. 5). In P1, triceps area decreased from 200.17 to 168.66 mm² (15.7% reduction). In P2, it declined from 56.02 to 27.5 mm² (50.9% reduction), with a mean reduction of 33% across both participants. CoG shifts were 2.87 mm (antero-superior) in P1 and 2.93 mm (antero-inferior) in P2 (mean 2.9 mm), consistent with a direct neuromodulatory effect at the stimulation site (Table 3). Biceps and deltoid representations showed a similar direction of change to triceps in both hemispheres (area compression from baseline to follow-up), but with visibly less pronounced compression and a less consistent CoG shift pattern than triceps, most evident on visual inspection of the unaffected hemisphere maps (Fig. 5), where only a slight increase in compression and focality toward the CoG was observed across all three muscles (Table 4).

**Table 3:** Triceps representation in the unaffected (right) hemisphere

Parameter	P1 (6 months post-amputation)	P2 (18 months post-amputation)	Mean
Baseline Area (mm²)	200.17	56.02	128
Follow-Up Area (mm²)	168.66	27.50	98
Area Reduction (mm²)	31.51	28.52	30
Area Reduction (%)	15.74	50.91	33
CoG Shift Magnitude (mm)	2.87 (antero-superior)	2.93 (antero-inferior)	2.90

*CoG: center of gravity; mm²: square millimeters*

**Table 4:** Resting motor threshold by hemisphere, participant, and session

Parameter	P1 unaffected (right)	P1 affected (left)	P2 unaffected (right)	P2 affected (left)
Baseline RMT (%MSO)	55	66	33	38
Follow-up RMT (%MSO)	62	67	34	33

*RMT: resting motor threshold*,* expressed as %MSO (percentage of maximum stimulator output); values reflect the triceps hotspot RMT*,* the primary outcome muscle and rTMS target*

#### Interhemispheric comparison: rebalancing of cortical asymmetry

**Fig. 6:**
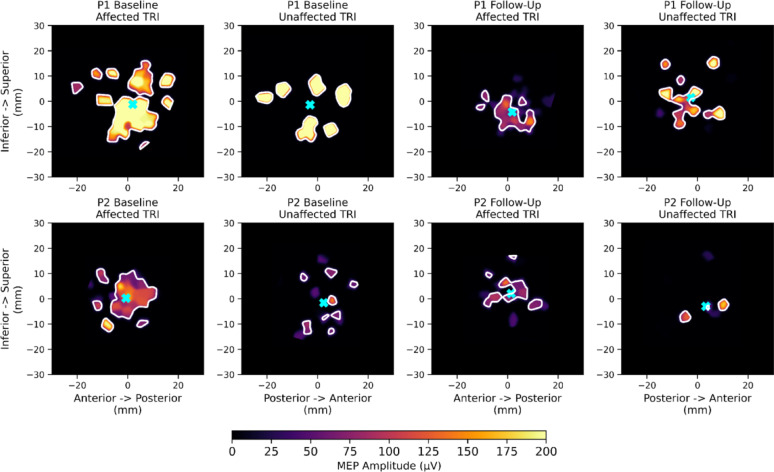
Interhemispheric comparison of triceps activation maps. Affected and unaffected hemisphere triceps maps displayed side-by-side for each participant at baseline and follow-up. Convergence of cortical excitability reflects normalization of interhemispheric asymmetry after rTMS

**Fig. 7:**
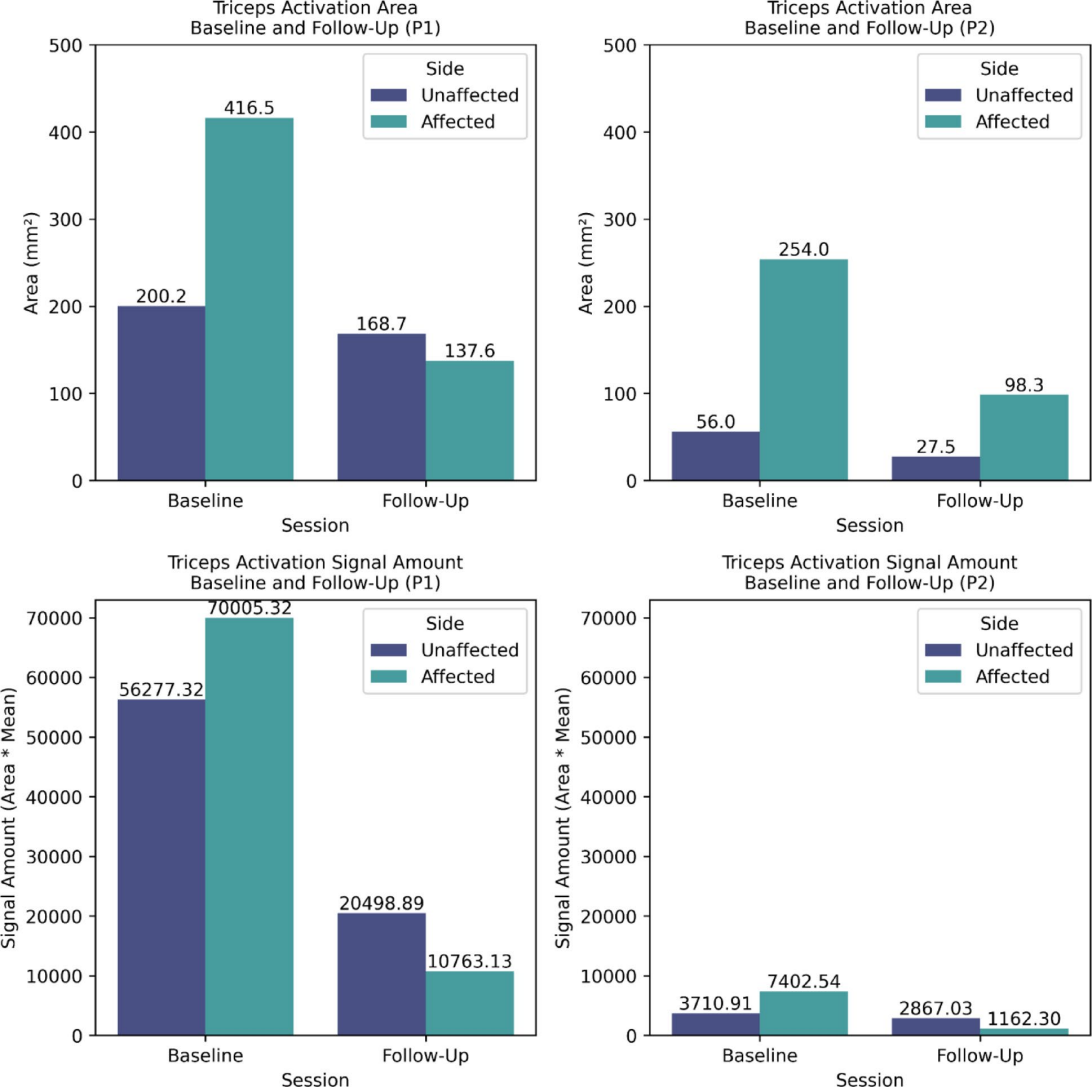
Triceps motor representation parameters by hemisphere and session display increased similarity in follow-up compared to baseline. Activation area (mm²) and signal amount (mean MEP amplitude × area) for P1 and P2 at baseline and follow-up. *Navy*: Unaffected hemisphere; *Teal*: Affected hemisphere. *MEP: *Motor evoked potential;* mm²: *Square millimeters;* P1: *Participant 1;* P2: *Participant 2

At baseline, both participants showed pronounced interhemispheric asymmetry in triceps motor representation. The affected hemisphere area was 2.08-fold larger than the unaffected in P1 (absolute difference: 216 mm²) and 4.53-fold larger in P2 (absolute difference: 197 mm²). Following rTMS, these interhemispheric differences decreased dramatically: to 31 mm² in P1 and 70 mm² in P2 (Fig. 6). Absolute differences in signal amount similarly decreased from 13,727 to 9,735 in P1 and from 3,691 to 1,704 in P2 (Fig. 7). These reductions in both spatial extent and signal amount occurred in parallel with the clinically meaningful pain reductions reported above.

## Discussion

This study provides novel neurophysiological evidence that inhibitory rTMS targeting the ipsilateral M1 in persons with upper-limb amputation produces clinically meaningful PLP reduction alongside bilateral cortical reorganization characterized by normalization of interhemispheric motor map asymmetry. Two key original contributions emerge: first, the clinical demonstration that ipsilateral inhibitory stimulation yields sustained analgesia comparable in magnitude to the best reported outcomes for conventional contralateral rTMS protocols; second, the introduction of a quantitative bilateral nTMS mapping pipeline — incorporating PCA-based spatial projection, Gaussian kernel heatmaps, and bilateral CoG and area analyses — that for the first time tracks longitudinal interhemispheric cortical dynamics in response to neuromodulation in individuals with limb amputation.

### Clinical efficacy and comparison with existing evidence

The PLP reductions observed here (5.22 VAS points in P1; 2.28 points in P2) meet or exceed IMMPACT thresholds for clinically meaningful change [25] and compare favorably with effect sizes reported in recent systematic reviews of rTMS for PLP [21, 22, 26]. Our results extend the single published case report of ipsilateral rTMS for PLP [23], which reported sustained pain reduction but offered no mechanistic cortical data. Critically, the sustained pain relief across a five-week follow-up achieved with only 2 (P1) and 5 (P2) rTMS sessions highlights the potential efficiency of ipsilateral stimulation as a therapeutic strategy.

The greater pain reduction in P1 (6 months post-amputation) compared with P2 (18 months) despite fewer sessions raises the mechanistically interesting possibility that ipsilateral rTMS is most effective during a window of heightened cortical plasticity in the subacute post-amputation period. Prior work has noted that the extent and direction of cortical remapping depends on time since amputation [27]: in a cohort of 62 unilateral traumatic lower-limb amputees with PLP, Pacheco-Barrios et al. found a disorganized, anteriorly shifted hand-area center of gravity and reduced grey matter volume in the affected hemisphere, with this structural asymmetry appearing to attenuate with greater time since amputation. This parallels our own more pronounced baseline reorganization and greater analgesic response in the more recently amputated participant (P1), and the contrasting trajectories of our two participants underscore post-amputation chronicity as a potentially important moderator variable for future trials.

### Callosal disinhibition as the underlying mechanism

The interhemispheric imbalance hypothesis offers a plausible mechanistic framework for the observed effects. Under physiological conditions, transcallosal inhibitory circuits maintain dynamic equilibrium between M1 regions. Following unilateral amputation, permanent deafferentation is thought to disrupt this balance, altering interhemispheric excitability in a manner that manifests as expanded cortical representations of residual limb muscles in the affected hemisphere (consistent with our baseline observations) [28] alongside bilateral microstructural and functional changes in transcallosal connectivity [13, 14]. We note that IHI in the strict neurophysiological sense refers to a paired-pulse transcranial magnetic stimulation measure of transcallosal inhibitory drive, which was not directly assessed in this study; we therefore use the broader term interhemispheric excitability balance when referring to the pattern inferred from our single-pulse mapping data.

Low-frequency (1 Hz) rTMS reliably reduces excitability at the stimulated site [17, 29]. When applied to the unaffected hemisphere, this inhibition is hypothesized to propagate via transcallosal pathways, reducing excessive inhibitory drive toward the deafferented hemisphere and thereby facilitating its adaptive reorganization — the callosal disinhibition hypothesis. This principle is well-established in stroke rehabilitation [18, 20] and has been demonstrated in a randomized trial of postoperative paresis [19]. The bilateral and directionally convergent nature of the post-intervention changes — area compression in both hemispheres with greater proportional effects in the affected hemisphere — is consistent with what callosal disinhibition would predict, although we did not directly measure interhemispheric inhibition and cannot exclude alternative explanations such as spontaneous change or non-specific effects of repeated stimulation. The modest CoG shifts (mean 3.28 mm) indicate normalization through focal map consolidation rather than topographic redistribution. The observed rebalancing echoes phenomena seen in mirror therapy, where PLP alleviation accompanies reversal of cortical reorganization [30].

### Cortical reorganization: relation to existing models

Our findings are most consistent with the classical maladaptive plasticity model [9–11]: both participants exhibited enlarged residual limb motor maps in the affected M1 at baseline, echoing prior observations by Karl et al. [31], Lotze et al. [32], and Raffin et al. [33]. The post-intervention normalization of these asymmetries paralleled the reduction in pain. The concurrent changes in the primary somatosensory cortex (S1), while not directly mapped here, may contribute to the pain reduction via somatosensory-motor network interactions and warrant investigation in future studies. The persistent representation model [12, 34] instead attributes chronic PLP to preserved structure and function of the former hand cortex together with reduced functional connectivity between this territory and the broader sensorimotor network, rather than to invasion by neighboring body-part representations; our design, focused on cortical area, amplitude, and CoG rather than functional connectivity, is not positioned to directly adjudicate between these frameworks, but the enlarged baseline motor maps observed here align more closely with the classical maladaptive plasticity account. This model, together with the heterogeneity of reorganization findings documented by Gunduz et al. [35] are not mutually exclusive with our results; our study adds a novel longitudinal bilateral neurophysiological perspective complementary to predominantly MRI-based evidence.

### The novel bilateral nTMS mapping pipeline

A central methodological contribution of this study is the PCA-based bilateral nTMS motor mapping pipeline. The present approach provides several advantages over prior single-hemisphere qualitative mapping in persons with amputation: (1) PCA-based 2D projection preserves metric spatial relationships across sessions; (2) Gaussian kernel heatmaps provide spatially continuous, amplitude-weighted representations of cortical motor output; (3) bilateral simultaneous mapping with identical protocols enables direct interhemispheric comparisons; and (4) signal amount captures both spatial extent and amplitude of cortical output in a single composite metric. This pipeline is fully replicable with standard Nexstim NBS data exports and provides the quantitative infrastructure needed for sample size calculations in future sham-controlled randomized trials.

### Limitations

The sample size of *N* = 2 reflects the intensive longitudinal neurophysiological characterization design, which is inherently suited to small, deeply phenotyped cohorts analogous to established single-case cortical mapping methodology. This enables mechanistic depth not achievable in larger heterogeneous cohorts but limits statistical generalizability. The absence of a sham stimulation condition precludes definitive attribution of cortical changes to rTMS versus spontaneous processes; however, area reductions of ≥ 60% bilaterally substantially exceed reported test-retest variability of nTMS motor mapping, lending confidence to a genuine rTMS-driven neurophysiological effect. The five-week follow-up does not address long-term durability. The differential number of sessions between participants was hypothesis-driven rather than randomized, limiting direct inter-participants comparisons. Given the sample size of *N* = 2 and the absence of direct paired-pulse IHI measurement, the callosal disinhibition interpretation offered here is hypothesis-generating rather than confirmatory and should be tested directly in future controlled studies. Our Gaussian kernel regression (bandwidth h = 1 mm) constructs the smoothed activation surface from all recorded MEP amplitudes, not only those exceeding 50 µV; the 50 µV iso-contour is applied only afterward to delineate the reported area boundary, comparable to threshold-based area methods used in other nTMS mapping studies [36]. A formal sensitivity analysis varying this threshold was not performed here but represents a useful methodological extension for future work.

## Conclusions

This exploratory study provides novel neurophysiological evidence suggesting that inhibitory rTMS applied to the ipsilateral M1 may reduce PLP while normalizing interhemispheric motor cortical asymmetry. The convergent reduction of cortical representation differences between hemispheres — alongside sustained clinical pain relief — is consistent with a callosal disinhibition mechanism, which should be directly tested in future studies incorporating paired-pulse interhemispheric inhibition measurements, and positions interhemispheric rebalancing as a candidate therapeutic target for PLP neuromodulation. The novel quantitative bilateral nTMS mapping pipeline introduced here provides a replicable methodological framework for future sham-controlled trials. Together, these findings challenge the prevailing paradigm of exclusive contralateral cortex stimulation and open a new mechanistic avenue for the treatment of post-amputation pain.

## Data Availability

The datasets generated and analysed during the current study, including individual participant VAS pain scores and nTMS motor mapping data, are not publicly available due to patient data privacy and institutional data protection regulations. The data are stored on secured servers of the Technical University of Munich (TUM) and are available from the corresponding author on reasonable request, subject to applicable data protection requirements and institutional approval.

## Abbreviations

BIC: Biceps brachii
CoG: Center of gravity
DEL: Deltoid
EMG: Electromyographic/electromyography
IHI: Interhemispheric inhibition
IMMPACT: Initiative on Methods, Measurement, and Pain Assessment in Clinical Trials
M1: Primary motor cortex
MEP: Motor evoked potential
MRI: Magnetic resonance imaging
nTMS: Navigated transcranial magnetic stimulation
P1: Participant 1
P2: Participant 2
PCA: Principal component analysis
PLP: Phantom limb pain
RMT: Resting motor threshold
rTMS: Repetitive transcranial magnetic stimulation
S1: Primary somatosensory cortex
TMS: Transcranial magnetic stimulation
TRI: Triceps brachii
VAS: Visual Analog Scale
